# High quality colonoscopy: using textbook process as a composite quality measure

**DOI:** 10.1055/a-2069-6588

**Published:** 2023-05-22

**Authors:** Karlijn J. Nass, Sascha C. van Doorn, Paul Fockens, Colin J. Rees, Maria Pellisé, Manon van der Vlugt, Evelien Dekker

**Affiliations:** 1 Department of Gastroenterology and Hepatology, Research Institute Amsterdam Gastroenterology and Metabolism, Amsterdam University Medical Center, Amsterdam, The Netherlands; 2 Department of Gastroenterology and Hepatology, Flevo Hospital, Almere, The Netherlands; 3 Department of Gastroenterology, Bergman Clinics, Amsterdam, The Netherlands; 4 Population Health Sciences Institute, Newcastle University, Newcastle-upon-Tyne, UK; 5 Gastroenterology Department, Endoscopy Unit, ICMDiM, Hospital Clinic, CIBEREHD, IDIBAPS, University of Barcelona, Catalonia, Spain

## Abstract

**Background** High quality colonoscopy is fundamental to good patient outcomes. “Textbook outcome” has proven to be a feasible multidimensional measure for quality assurance between surgical centers. In this study, we sought to establish the “textbook process” (TP) as a new composite measure for the optimal colonoscopy process and assessed how frequently TP was attained in clinical practice and the variation in TP between endoscopists.

**Methods** To reach consensus on the definition of TP, international expert endoscopists completed a modified Delphi consensus process. The achievement of TP was then applied to clinical practice. Prospectively collected data in two endoscopy services were retrospectively evaluated. Data on colonoscopies performed for symptoms or surveillance between 1 January 2018 and 1 August 2021 were analyzed.

**Results** The Delphi consensus process was completed by 20 of 27 invited experts (74.1 %). TP was defined as a colonoscopy fulfilling the following items: explicit colonoscopy indication; successful cecal intubation; adequate bowel preparation; adequate withdrawal time; acceptable patient comfort score; provision of post-polypectomy surveillance recommendations in line with guidelines; and the absence of the use of reversal agents, early adverse events, readmission, and mortality. In the two endoscopy services studied, TP was achieved in 5962/8227 colonoscopies (72.5 %). Of 48 endoscopists performing colonoscopy, attainment of TP varied significantly, ranging per endoscopist from 41.0 % to 89.1 %.

**Conclusion** This study proposes a new composite measure for colonoscopy, namely “textbook process.” TP gives a comprehensive summary of performance and demonstrates significant variation between endoscopists, illustrating the potential benefit of TP as a measure in future quality assessment programs.

## Introduction

High quality is at the core of healthcare provision. Clinical audit can be a valuable tool in healthcare to improve the performance of an individual doctor, department, or hospital, thereby contributing to improvement in the quality of care 1 2 . For colonoscopy, such quality measures exist and have shown their benefits in relation to patients’ risks of postcolonoscopy colorectal cancer (PCCRC). For example, the adenoma detection rate (ADR) has been found to be inversely associated with the occurrence of PCCRC 3 4 . Audit and feedback on colonoscopy quality measures resulted in improved performance of these measures 5 . Data on single quality indicators do not however reflect the complete process of a colonoscopy and may not reliably measure the overall quality of this procedure.

Composite measures combine several aspects of quality for specific procedures, resulting in an all-or-none measurement. Such composite measures may provide a better and potentially more stable reflection of overall quality, as opposed to a single outcome indicator. Moreover, all-or-none measurements may be more suitable for quality monitoring owing to their being a more sensitive reflection of performance 6 . All-or-none performance measures have been successfully introduced for the ideal outcome of surgery and have been termed “textbook outcome.” This has been defined for several surgical procedures 7 8 9 10 11 12 13 14 . For example, textbook outcome after pancreatic surgery includes the absence of postoperative pancreatic fistulas, bile leak, post-pancreatectomy hemorrhage, severe adverse events (AEs), readmission, and in-hospital mortality 13 . Textbook outcome has been demonstrated to be a feasible and useful parameter in the surgical field for comparison of performance between hospitals 7 8 9 10 11 12 13 .

The result of an all-or-none measurement may provide a better indication of overall quality and provide a better opportunity for quality improvement when compared with data on a single quality indicator. With single quality indicators, compliance of > 90 % is often achieved, leading to limited space for further improvement, which could temper the motivation for changes in clinical practice. If there is more room for improvement, this will more likely lead to quality improvement initiatives 6 .

In this study, we aimed to investigate an “all-or-none” measure for colonoscopy. We used a modified Delphi consensus process to propose a definition for the optimal process of colonoscopy: the “textbook process” (TP). TP includes multiple components that, when all achieved, could represent the ideal process for colonoscopy. All of the included components should be assessable per colonoscopy. After defining TP, we assessed the achievement of TP in two endoscopy services and measured the variation between endoscopists. The ADR is regarded as the most important contemporaneous measure of colonoscopy quality 15 16 17 , but it is not assessed at an individual colonoscopy level. Given the established importance of the ADR as a quality marker, we evaluated the potential additive value of TP by assessing the correlation between the achievement of TP and the ADR.

## Methods

### Definition of textbook process

A modified Delphi consensus process on potential items for the TP was performed 18 . At total of 27 expert endoscopists from different countries in Europe were invited to complete an online questionnaire. These experts were selected based on their participation in international guidelines focusing on the quality of colonoscopy and research in this field. The survey consisted of eight sections with a total of 14 items, each of which were rated on a five-point Likert scale based on agreement or disagreement with that particular item being a requirement for TP. These items were based on the recommendations in the ESGE Guideline on performance measures for lower gastrointestinal endoscopy 15 .

The proposed definition of TP was designed for diagnostic and surveillance colonoscopies. It did not aim to cover therapeutic colonoscopies, because our definition of therapeutic colonoscopies is that these are colonoscopies specifically planned for the removal of previously diagnosed colorectal lesion(s) or planned for dilations (i. e. piecemeal endoscopic mucosal resection for a large adenoma, endoscopic submucosal dissection, or endoscopic full-thickness resection). As therapeutic colonoscopies serve a different purpose, TP was not reviewed in these colonoscopies. For example, when a colonoscopy is performed for endoscopic mucosal resection of a large lesion in the transverse colon, the intention does not always include reaching the cecum.

Initially, the potential items for the definition of TP were: explicit indication for colonoscopy documented in the endoscopy report; successful cecal intubation; adequate bowel preparation; adequate withdrawal time; acceptable patient comfort (Gloucester Comfort Scale (GCS) 1–2 or GCS 1–3); no higher sedation dose than the median dose within the monitored population; no use of reversal agents; no early AEs; no all-cause or colonoscopy-specific readmissions; no all-cause or colonoscopy-specific mortality; and an appropriate post-polypectomy surveillance recommendation (as defined by published guidance). An agreement rate of ≥ 80 % per item by Delphi process was considered to be consensus and the item was included in the definition of TP. When, on a particular item, consensus was not reached, this item was reviewed in light of suggestions and comments, and adjusted if required. If 80 % agreement was not reached after a maximum of three rounds, consensus was defined as having been reached if > 50 % of the experts voted in favor and < 20 % voted against this specific item 18 . Failure to meet these criteria resulted in the item being discarded from the definition.

### Assessment of textbook process

This retrospective study was conducted with prospectively collected data from the Bergman Clinics, location Amsterdam and Bilthoven, The Netherlands. Essential patient and endoscopy data were prospectively collected in the endoscopy reporting system, Endobase (Olympus, Tokyo, Japan), and histopathology data were added directly after histopathological evaluation. All data were automatically extracted from the reporting system into one large dataset. As part of standard care, the post-polypectomy surveillance recommendation was recorded in the electronic patient chart. These data could be automatically extracted in one endoscopy service. No individual patient records were reviewed for this study.

Colonoscopies performed between 1 January 2018 and 1 August 2021 were analyzed. TP was assessed in colonoscopies performed for the indication of symptoms and for surveillance colonoscopies. Procedures were excluded when patients were aged < 18 years. Data about AEs are recorded in the local and national AE registry 19 . Data from these registries were linked to the dataset. The obtained dataset was anonymized and provided for research purposes. As anonymized data were used, no ethical approval was required by the Institutional Review Boards.

Endoscopists were both gastroenterologists and supervised gastrointestinal fellows in training. The two endoscopy services closely collaborate with two academic hospitals. Gastroenterologists and fellows from the academic hospitals work rotating shifts in the endoscopy services from which data were used in this study.

### Definitions for the assessment of TP

The main outcome for this study was the achievement of TP in diagnostic and surveillance colonoscopies. The achievement of TP was analyzed per colonoscopy. When one of the items of TP was not achieved, the colonoscopy was considered not to have achieved TP. When data on an item were missing, this item was considered as not achieved and consequently TP was not achieved.

Definitions of the individual TP items are described in Table 1 . The quality of bowel preparation was assessed by the validated Boston Bowel Preparation Scale 20 21 . Patient comfort during colonoscopy was reported as the nurse-assessed modified GCS 22 . Only colonoscopies in which no polypectomy or biopsy was required were included when calculating the withdrawal time, to remove the potential bias of including the additional time taken for therapeutic interventions during withdrawal. In this study, future surveillance recommendations were checked for their appropriateness once histopathology had been obtained. An adequate surveillance recommendation was defined as a recommendation in line with the Dutch guideline on colonoscopy surveillance 23 . The ADR was defined as the proportion of colonoscopies where at least one adenoma was detected, based on histopathology.

**Table 1:** Definition of textbook process for diagnostic colonoscopy – the required items were selected after achieving consensus in a modified Delphi process.

	Items	Definition	Agreement rate
1	Explicit indication	The colonoscopy report includes an explicit indication for the procedure	100 %
2	Successful cecal intubation	Successful cecal intubation, meaning complete visualization of the whole cecum and its landmarks, which is described in the report and documented by photo or video	100 %
3	Adequate bowel preparation	Adequate bowel preparation, which is defined as a Boston Bowel Preparation Scale of at least 2 per segment	100 %
4	Adequate withdrawal time	Adequate withdrawal time, which is defined as a minimum of 6 minutes spent during withdrawal of the endoscope from the cecum into the anus in a negative colonoscopy (without any intervention)	85 %
5	Acceptable patient comfort 1	An acceptable patient comfort score, defined as a modified Gloucester Comfort Scale of 1 or 2	75 % 2
6	No use of reversal agents	No need for use of reversal agents (such as naltrexone, flumazenil) during or after colonoscopy	75 % 2
7	No early AEs	No early AEs, which are defined as AEs that fulfill both of the following two criteria: (i) AE is diagnosed during the procedure or before discharge of the patient: AND (ii) AE resulted in either lengthening of the hospital stay, unscheduled additional colonoscopy, or an emergency intervention, including blood transfusion or surgery	90 %
8	No 14-day readmission	No all-cause 14-day readmission after colonoscopy	70 % 2
9	No 30-day mortality	No all-cause 30-day mortality	85 %
10	Adequate post-polypectomy surveillance recommendation	A post-polypectomy surveillance recommendation is given in line with national guidelines (based on histopathology)	95 %

AE, adverse event.

1 Patient comfort will not be taken into account as a requirement for textbook process if deep sedation (propofol or general anesthesia) is being used. It should be assessed only in patients with no sedation or conscious sedation.

2 More than 50 % of the experts voted in favor, < 20 % voted against this particular item in the third voting round.

### Statistical analysis

Non-normally distributed continuous variables are presented as the median and interquartile range (IQR). Categorical variables are expressed as numbers and percentages. TP was determined for every colonoscopy, according to the selected requirements through the survey. In per-endoscopist analyses, only data from endoscopists with experience of more than 20 colonoscopies in our study population were included. TP rates per endoscopist are presented in a funnel plot and effects are shown as a sequence of 95 %CIs. The correlation between the achievement of TP and the ADR was assessed by the Spearman rank correlation test. All statistical tests were two-sided at an α level of 0.05. All statistical analyses were performed using R statistical software, version 3.5.1 (R Foundation for Statistical Computing, Vienna, Austria; www.R-project.org/).

## Results

### Definition of textbook process

In total, 20 of the 27 invited expert endoscopists (74.1 %) completed all three rounds of the modified Delphi consensus. After three voting rounds, 10 items were accepted for the TP for colonoscopy ( Table 1 and **Table 1 s,** see online-only Supplementary material). TP was defined as a colonoscopy fulfilling the following requirements: explicit colonoscopy indication; successful cecal intubation; adequate bowel preparation; adequate withdrawal time; acceptable patient comfort score; post-polypectomy surveillance recommendation in line with current guidelines; and the absence of use of reversal agents, early AEs, all-cause readmission within 14 days after the procedure, and all-cause mortality within 30 days after the procedure. During the modified Delphi process, the experts did not reach consensus on the item focused on the sedation dose, so this was not included in the definition of TP.

### Study population characteristics

**Fig. 1:**
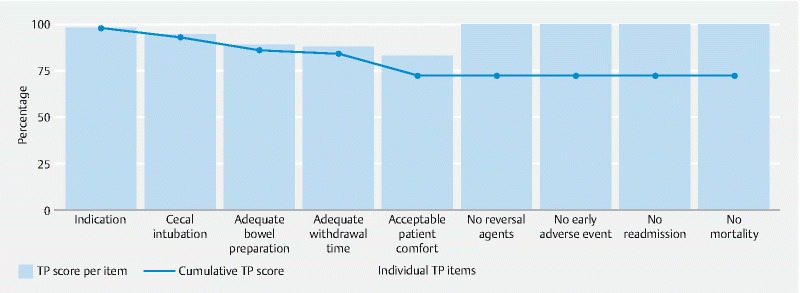
Rates for achievement of each individual item of the textbook process (TP) and cumulative rate for achievement of TP.

During the study period, 13 481 colonoscopies were performed. After exclusion, data from 8227 colonoscopies were available for analysis ( **Fig. 1 s** ). The median (IQR) age of patients undergoing colonoscopy was 61 (51–70) years and 51 % of the patients were women. An American Society of Anesthesiologists (ASA) score of II was most frequent among the included patients (n = 3919; 47.6 %). All colonoscopies were performed without sedation (n = 692; 8.4 %) or under conscious sedation. In the total study population, of those with the indication available, 5378 were performed for symptoms and 2708 for surveillance.

### Textbook process

The proportion of colonoscopies in which TP was achieved was 72.5 % (5962/8227). The individual rates per item and cumulative rates are shown in Fig. 1 . Acceptable patient comfort and an adequate withdrawal time in a negative colonoscopy were the individual TP items that were least commonly achieved (83.1 % and 87.9 %, respectively) ( **Table 2 s** ). Appropriate surveillance recommendation was not included as an item for TP in these analyses, as surveillance data were not available for both endoscopy services.

In a subanalysis, appropriate surveillance recommendation was included in the definition of TP using the colonoscopy data for only one endoscopy service. In this endoscopy service, appropriate surveillance recommendations, based on histopathology, were provided in 98.2 % of the colonoscopies for which surveillance recommendations were required (n = 3112). Based on this item, the proportion of colonoscopies in which TP was achieved was not significantly different (72.5 % in the total study population vs. 72.2 % when including surveillance recommendation; *P* = 0.71).

**Fig. 2:**
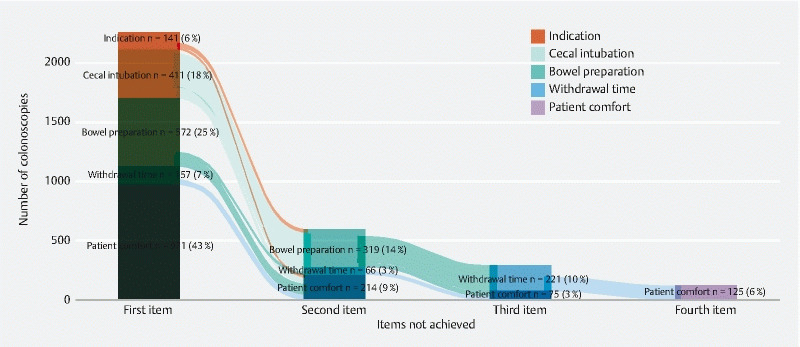
Combination of the textbook process items that were not achieved. The first column represents the first item that was not achieved; when more than one item was not achieved, the different combinations of items not achieved are shown in the remaining columns (groups of n < 50 are not shown in this figure).

Fig. 2 demonstrates which item was not achieved if only one item was not achieved, as well as combinations of items that were not achieved when more than one item was not achieved.

### Variation between endoscopists

**Fig. 3:**
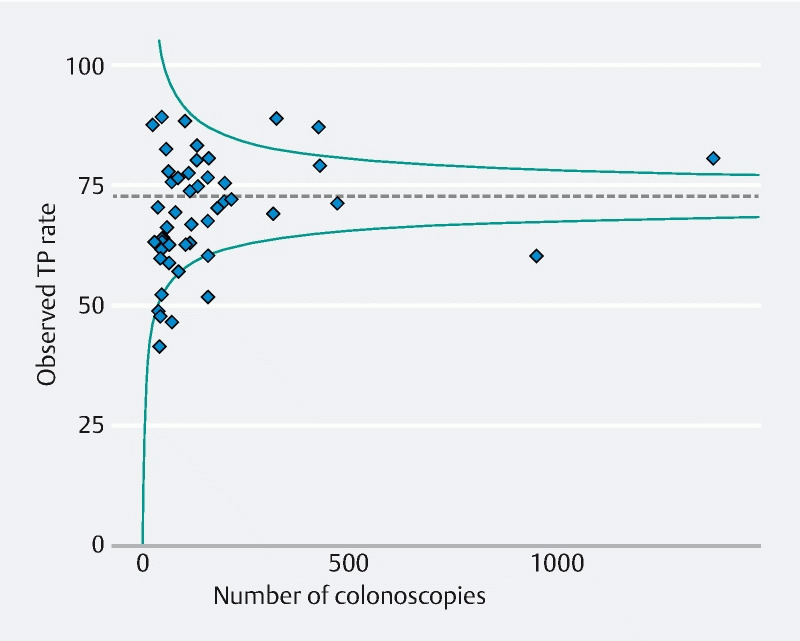
Variation in textbook process (TP) rates per endoscopist and relationship to number of colonoscopies performed, with benchmark (dotted line) and 95 %CIs (solid lines) shown.

During the study period, 48 endoscopists performed more than 20 colonoscopies for the study (median [IQR] 104 [49–158] colonoscopies per endoscopist). The achievement of TP per endoscopist varied between 41.0 % and 89.1 % (median [IQR] 69.7 % [62.2 %–77.6 %]) ( Fig. 3 ).

### Correlation of textbook process and adenoma detection rate

**Fig. 4:**
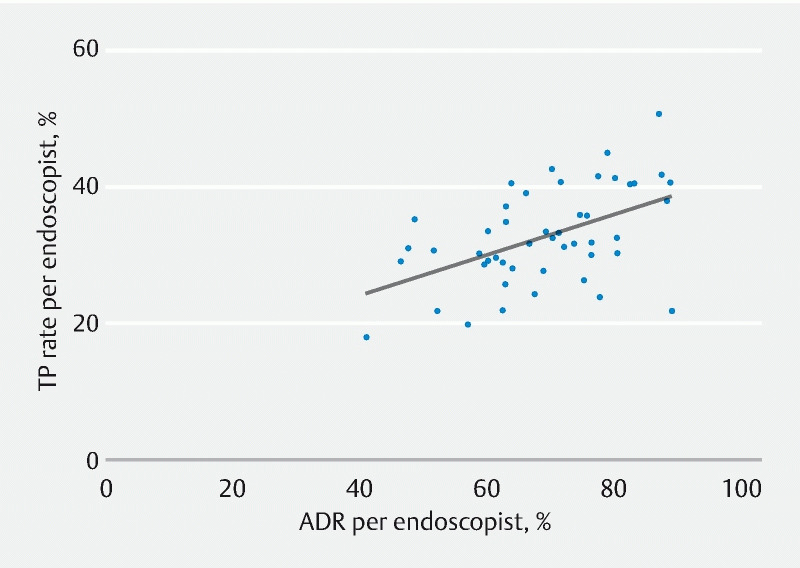
Correlation between the textbook process (TP) rate and the adenoma detection rate (ADR) per endoscopist (Spearman rank correlation coefficient, *r* = 0.48; *P* < 0.001).

The overall ADR was 34.1 %. The median (IQR) ADR per endoscopist was 31.7 % (28.8 %–38.1 %). TP showed a moderate correlation with the ADR ( *r* = 0.48; *P* < 0.001) ( Fig. 4 ).

### Subanalysis of textbook process without comfort score

In a subanalysis, the variable “acceptable patient comfort” was not taken into account. TP was achieved in 6928 colonoscopies (84.2 %) following this definition. Without the variable “acceptable patient comfort,” the achievement of TP varied between 41.0 % and 95.4 % per endoscopist.

## Discussion

We propose TP as a new composite measure for reporting the quality of colonoscopy. TP includes multiple quality items that, when they are all achieved, represent the ideal process of colonoscopy. In two endoscopy services, TP was achieved in 72.5 % of colonoscopies and the achievement of TP varied significantly between endoscopists. TP provides a comprehensive summary of performance, removing the bias that is possible in practice driven by the use of single quality indicators. We believe this illustrates the potential benefit of this measure in future quality assessment programs and that it could be used as an addition to the pre-existing performance measures 15 . As described earlier, single performance measures may not reflect the overall quality of colonoscopy. TP is more comprehensive and easier to measure, so it could therefore be used as a first screening before exploring other quality indicators in greater detail. When an endoscopist or endoscopy service underperforms on TP, the individual items in the TP could be analyzed in more detail, aiming ultimately to initiate targeted improvement processes. Therefore, the all-or-none measurement used for TP would encourage the evaluation of individual components instead of precluding them 6 .

To fully appreciate our findings, some limitations need to be addressed. First, the importance of structured and standardized endoscopy reporting to facilitate quality assessment has been underlined in this study. Structured and standardized endoscopy reporting systems and systematic reporting of AEs are prerequisites for the measurement of TP to be feasible in endoscopy practice 24 . In this study, the data were obtained directly from the endoscopy reporting system. When the structured and standardized fields were not recorded for a particular variable, this variable was missing in our data. Therefore, we may have underestimated the results of TP in this study. Endoscopists should be encouraged to use standardized and structured endoscopy reporting to reliably measure their performance. Nevertheless, when TP is included in quality assessment programs in the future, this might be an incentive to use standardized and structured reporting systems, as endoscopists and endoscopy services will ultimately be accredited and audited on these results.

Second, when no AE was registered in the national or local AE registry, we assumed no AE had occurred. It is theoretically possible that we underestimated the actual number of AEs in this study; however, much attention has been paid to complete registration for local quality purposes and local morbidity and mortality discussions. Third, owing to logistic issues, we had access to the surveillance data of only one of the two endoscopy services; however, a subanalysis including adequate surveillance recommendation in a subpopulation did not change our main results. Fourth, some TP items are usually monitored as a mean, calculated in a subset of colonoscopies instead of per colonoscopy. For example, it is recommended that withdrawal time is assessed by dividing the sum of the colonoscopy withdrawal times by the number of colonoscopies performed 15 . Nevertheless, all TP items were evaluated per colonoscopy in this study to assess TP per colonoscopy. Finally, the external validity of our results outside the Netherlands is not clear yet. Therefore, TP should be evaluated on a larger scale in different countries in future research projects.

Potential items for TP were adjusted or omitted based on the experts’ comments during the iterative rounds of the modified Delphi consensus process. Inclusion of all-cause or colonoscopy-specific AEs and mortality was extensively discussed during the Delphi process. AEs could be distinguished according to the cause. All-cause AEs are less sensitive for subjective interpretation because the relationship between a procedure and any AE is often speculative 25 ; however, monitoring the all-cause AE rate is an overestimation of the actual AE rate related to colonoscopy. The colonoscopy-specific AE rate is probably more accurate but, owing to subjective assessment of the relationship between the colonoscopy and the AE, probably more challenging to compare across endoscopists, services, and countries.

A parameter reflecting sedation practice by an individual endoscopist or endoscopy service as a requirement for TP was proposed in the first rounds of the Delphi process. Consensus was not reached however, most likely because of the wide variation in sedation practices across countries 26 27 28 29 . A recent study performed with data from the national fecal immunochemical test (FIT)-based CRC screening program showed relatively high dosage rates of sedation in the Netherlands as compared with other countries, such as the UK 26 30 . More research is needed on the effect of higher sedation dose on the quality and safety of colonoscopy. Additionally, cultural differences in sedation practice between different countries will make this a possibly less valuable item outside national registries.

The aim of the TP should not be to reach 100 %. For example, if an obstructing tumor is found, cecal intubation will not be possible and TP cannot be achieved. In our study, TP was achieved in 72.5 % of the colonoscopies; however, to set a reliable benchmark, TP should be measured on a larger scale.

Of all the items, adequate patient comfort was the factor least commonly achieved. The relatively high number of colonoscopies that did not achieve acceptable patient comfort was due to scoring GCS 3 (mild discomfort; 1033 /1247). During the Delphi process, mild discomfort (GCS 3) was also considered acceptable in the definition of TP, but was omitted during the iterative rounds; however, a GCS of 3 is considered acceptable in some literature 30 . Furthermore, there is a question as to whether the GCS is the optimal measure for assessing patient comfort. Recently, the Newcastle ENDOPREM has been introduced as a patient-reported experience measure for gastrointestinal endoscopy and seems promising 31 ; however, the feasibility of this measure for incorporation into composite quality measures is not yet known. When looking at the results of the individual TP items in this study, most items reached the proposed minimum standard (if defined) of the ESGE guideline on performance measures for lower gastrointestinal endoscopy 15 . Adequate bowel preparation rate (89.1 %) was the only item that did not reach the proposed minimum standard (90.0 %) 15 .

Several items of the TP were achieved in almost 100 % of the colonoscopies. When TP is implemented in daily practice, and during successive evaluations, if there is a lack of variety in some items, these items might be omitted in future versions of the TP.

Similarly to the studies about textbook outcome in the surgical field 7 8 9 10 11 12 13 , wide variation in the achievement of TP per endoscopist was seen in this study. Although potential differences in case-mix factors between endoscopists were not considered, this variability seems undesirable. The first step to evaluate this further would be to assess the individual items of TP per endoscopist. Quality assurance programs might improve the rates per endoscopist, especially for the low performers. Low performers seem to benefit the most from feedback interventions, as shown for the ADR in a recent systematic review 5 .

Future efforts should focus on the assessment of TP in larger populations and the use of TP for comparisons between services. In a subanalysis, TP was calculated without acceptable patient comfort in the definition, leading to higher TP rates. Nevertheless, considerable variation between endoscopists remained. Therefore, TP might still be considered a useful performance measure in countries where most colonoscopies are performed with deep sedation and where, therefore, the GCS cannot be assessed.

As the ADR cannot be assessed per individual colonoscopy, it could not be included in the TP. The TP includes multiple components that, when all achieved, could represent the ideal process for colonoscopy. The ADR is not measurable in this form, as it is not required to detect at least one adenoma in every colonoscopy. One could perform an ideal colonoscopy (i. e. reaching the cecum in a well-cleansed colon with little or no discomfort) without detecting an adenoma. Therefore, the ADR is not included in the definition of TP. Nevertheless, the ADR remains one of the most important performance measures in colonoscopy owing to its inverse association with the PCCRC 3 4 . In this study, TP showed a moderate correlation with the ADR. Furthermore, TP showed its additive value alongside monitoring the ADR alone. When looking at the vast majority of endoscopists who reached the recommended ADR cutoff of 25 % 15 , considerable variation in the achievement of TP still existed between endoscopists. Evaluation of the individual items of TP might identify targets for further quality improvement. Moreover, TP might have advantages in terms of feasibility compared with the ADR. Monitoring the ADR in daily practice has limitations, as histopathology is needed from each colonoscopy. A validated and accurate measure that does not require this evaluation might have significant advantages for continuous quality assessment in daily endoscopy.

In conclusion, TP gives a comprehensive summary of performance and varies considerably between endoscopists. TP should be considered as one of the performance measures in future quality assessment programs to get insight into the overall quality of colonoscopy. Future studies should further validate this new composite performance measure to set a benchmark for TP.

## Table 1 s, 2 s, Fig. 1 s

Supplementary material Zusatzmaterial
