# Supplementary material for: High quality colonoscopy: using textbook process as a composite quality measure
Source: Endoscopy. 2023 May 22;55(9):812–9. doi: 10.1055/a-2069-6588 (PMC10465239; doi:10.1055/a-2069-6588)
Supplement: Supplementary file 1 — Supplementary material [file 22048supmat_10-1055-a-2069-6588.pdf]

Supplementary material

High quality colonoscopy: using textbook process as a composite quality measure

Karlijn J. Nass, Sascha C. van Doorn, Paul Fockens, Colin J. Rees, Maria Pellisé,

Manon van der Vlugt, Evelien Dekker

Table 1s. *Voting rates for different Textbook Process items in three rounds during the Modified Delphi Process*

**Textbook process items**	**Round 3 (20 responses)**	**Round 2 (20 responses)**	**Round 1 (23 responses)**
Explicit indication in the report	100%	100%	NA
Cecal intubation	100%	100%	100%
Adequate bowel preparation	100%	100%	100%
Adequate withdrawal time	85%	75%	83%
Acceptable patient comfort GCS 1-2	75%^	85%	65%
~~Acceptable patient comfort GCS 1-3*~~	NA	NA	52%
~~Sedation dose !> median dose*~~	NA	35%	35%
No reversal agents	75%^	75%	65%
No early adverse events	90%	80%	74%
No all-cause 14-day readmission	70%^	55%	27%
~~No colonoscopy-specific 30-day readmission*~~	NA	70%	81%
No all-cause 30-day mortality	85%	70%	35%
~~No colonoscopy-specific 30-day mortality*~~	NA	70%	73%
Adequate post-polypectomy surveillance recommendation	95%	95%	87%

** Items that were omitted during the subsequent rounds of the modified Delphi process.*
^ More than 50 % of the experts voted in favor, < 20 % voted against this particular item in the third voting round
Abbreviations: GCS, modified Gloucester Comfort Scale; NA, not applicable

Supplementary material

***Fig. 1s**: Inclusions and exclusions within the study.*

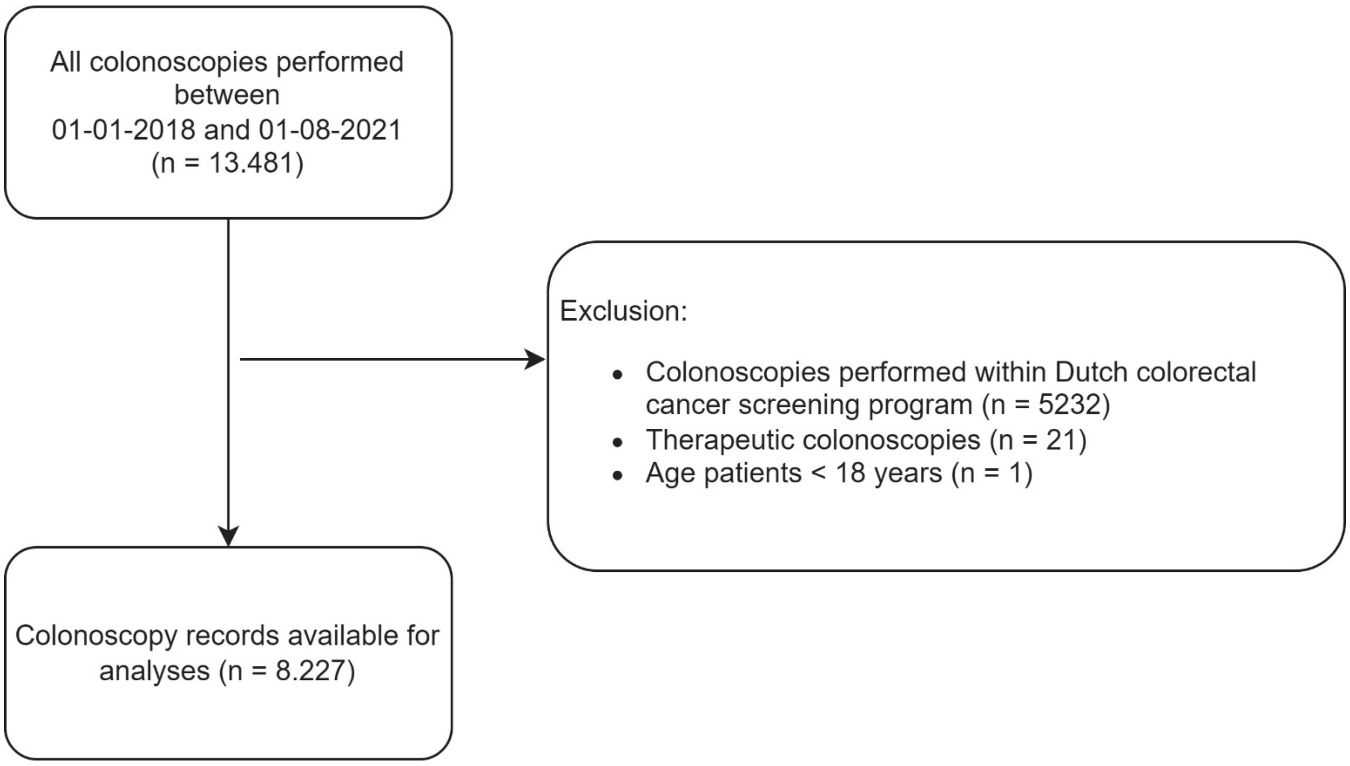

Supplementary material

Table 2s. *The percentages of the individual items included in Textbook Process and the conditional percentages after adding one of the items after each other for Textbook Process.*

Textbook Process items	Score per item	Cumulative score
1. Explicit indication in the report	98.3%	98.3%
2. Cecal intubation	94.9%	93.3%
3. Adequate bowel preparation	89.1%	86.3%
4. Adequate withdrawal time (n = 3700*)	87.9%	84.4%
5. Acceptable patient comfort	83.1%	72.6%
6. No reversal agents	99.9%	72.5%
7. No early adverse events	100%	72.5%
8. No all-cause 14-day readmission	99.9%	72.5%
9. No all-cause 30-day mortality	100%	72.5%

** Different denominator, as the adequacy of the withdrawal time is only determined in negative colonoscopies*
